# Total Water–Soluble Flavonoids From *Lithocarpus litseifolius* (Hance) Chun (Sweet Tea) Improve Glucose Homeostasis Through Multitarget Signalling in GDM Mice

**DOI:** 10.1155/2024/1518080

**Published:** 2024-11-13

**Authors:** Junfei Xu, Fenfang Zhang, Huanhuan Li, Pan Li, Junying Zeng, Xianjin Wu, Rong Zhou, Chunyan Yang, Juzuo Zhang

**Affiliations:** ^1^College of Biological and Food Engineering/Key Laboratory of Research and Utilization of Ethnicinal Plant Resources of Hunan Province/Hunan Provincial Higher Education Key Laboratory of Intensive Processing Research on Mountain Ecological Food, Huaihua University, Huaihua 418000, China; ^2^Department of Obstetrics and Gynecology, Huaihua Second People's Hospital/Huaihua Cancer Hospital, Huaihua 418000, China

**Keywords:** GDM, glucose homeostasis, multiple targets, total water–soluble flavonoids

## Abstract

**Background:** The oral safety of *Lithocarpus litseifolius* (Hance) Chun (sweet tea) that has antihyperglycemic potential has been verified. However, its specific application and action mechanism in the treatment of gestational diabetes mellitus (GDM) are still unclear.

**Methods:** Total water–soluble flavonoids extracted from *L. litseifolius* (Hance) Chun (sweet tea) were applied to GDM mice. The glucose tolerance, insulin sensitivity, and histopathology of the GDM mice were evaluated through an intraperitoneal glucose tolerance test (IPGTT), an intraperitoneal insulin tolerance test (IPITT), and histochemistry. The possible mechanism was analysed through network pharmacology.

**Results:** Compared with those in GDM model mice (MD group), blood glucose levels indicating both glucose tolerance and insulin sensitivity were improved in GDM mice treated with total water–soluble flavonoids (LLHC group) but were greater than those in normal control mice (NC group). The number of apoptotic liver cells was significantly lower in the LLHC group than in the MD group, but greater than that in the NC group. Multiple targets and signalling pathways that were acted by eight main active ingredients were involved in the process by which total water–soluble flavonoids protect against GDM. The main mechanism involved quercetin (10 targets) and luteolin (8 targets), which acted on the effector target of GAA through six main signalling pathways around the AKT1 core axis.

**Conclusion:** Oral administration of total water–soluble flavonoids can alleviate glucose intolerance and insulin resistance via the inhibition of liver cell apoptosis. The main active ingredients act on GAA through the signalling pathways of the AKT1 core axis.

## 1. Introduction

Gestational diabetes mellitus (GDM) seriously affects the short- and long-term health of pregnant women and their offspring [1, 2]. Moreover, there has been a dramatic increase in the incidence of GDM [3]. The identification of efficient methods for GDM prevention and treatment is important and urgent. Previous studies have shown that relatively short durations and impaired insulin use are the leading causes of GDM occurrence [4]. Therefore, supplementation with insulin and improvements in sensitivity to insulin have become the main directions of clinical treatment.

Several efficient medicines, such as insulin, metformin, and glyburide, have been applied for GDM treatment [5, 6]. However, in many countries, only insulin is prescribed in GDM treatment [7]. Moreover, insulin treatment requires multiple injections and does not fully protect offspring from metabolic disorders, which is still difficult and unfriendly for patients receiving long-term medication and pregnant mothers [8–10]. Therefore, the search for safe and convenient antidiabetic drugs for women with GDM should be increased.

Oral glucose–lowering drugs have attracted increasing amounts of attention because they are noninvasively delivered to patients and are less expensive [11, 12]. Metformin and sulfonylureas, as oral glucose-lowering drugs, were widely clinically approved for the treatment of obesity and type 2 diabetes [13, 14]. However, the side effects of presenting some risks for foetal growth and development are still major obstacles in GDM treatment [13, 15–17]. It must be considered that anti-GDM drugs are friendly to the mother and safe for the mother and foetus. Considering the drug resistance, side effects, and unknown toxicology of these antidiabetic medicines, it is necessary and urgent to explore new anti-GDM drugs.

Like metformin, which was discovered in *Aegilops*, traditional Chinese medicine, a treasure that is rich in drug resources and prescriptions, may store many anti-GDM drugs. The use of traditional Chinese medicine for controlling GDM has achieved good results [18, 19]. Therefore, continuing investigations are necessary and valuable. On the basis of this idea, we identified *L. litseifolius* (Hance) Chun leaves, as a dietary and medicinal material that is processed into *L. litseifolius* (Hance) Chun (sweet tea), whose ability to intervene in diabetes, hypertension, and obesity has been recorded in the “Compendium of Materia Medica” and “National Compilation of Chinese Herbal Medicine”. Some studies have also shown that consuming *L. litseifolius* (Hance) Chun (sweet tea) has many health benefits, such as antihyperglycemic, anti-inflammatory, antioxidant, and anticancer effects [20–23]. More importantly, our previous study elucidated the main composition of total water–soluble flavonoids from *L. litseifolius* (Hance) Chun (sweet tea) and demonstrated their oral safety [24]. This evidence of its safety and antidiabetic potential suggests that total water–soluble flavonoids could be used as a basis for GDM treatment. In this study, a GDM mouse model was generated by a combination of high-fat diet (HFD) induction and intraperitoneal injection of streptozocin (STZ). Total water–soluble flavonoids were applied to GDM model mice through intragastric administration. The physiological and pathological states of the model mice were investigated through an intraperitoneal glucose tolerance test (IPGTT), an intraperitoneal insulin tolerance test (IPITT), and dissection and tissue microsection analysis. The possible mechanism was further investigated through network pharmacology. This study provides a foundation for subsequent research on the exact mechanism and pharmaceutical industrial application of *L. litseifolius* (Hance) Chun (sweet tea).

## 2. Materials and Methods

### 2.1. Preparation of Total Water–Soluble Flavonoids

Total water–soluble flavonoids were separated from *L. litseifolius* (Hance) Chun (sweet tea) via ultrasonic-assisted water extraction according to our previous method [24]. Briefly, a fine powder of *L. litseifolius* (Hance) Chun (sweet tea) was obtained after pulverization and sieving and mixed with cold deionized water for the extraction of total water–soluble flavonoids at a liquid/solid ratio of 33:1 mL/g, a temperature of 61°C, a microwave power of 634 W, and a microwave time of 165 s. The extracts were filtered through a 0.45 *μ*m filter with a vacuum pump after centrifugation at 5000 rpm for total water–soluble flavonoids. After content detection, total water–soluble flavonoids were concentrated to the desired concentration with a rotary evaporator for the following experiments.

### 2.2. Development of the GDM Mouse Model and Intervention

**Figure 1:**
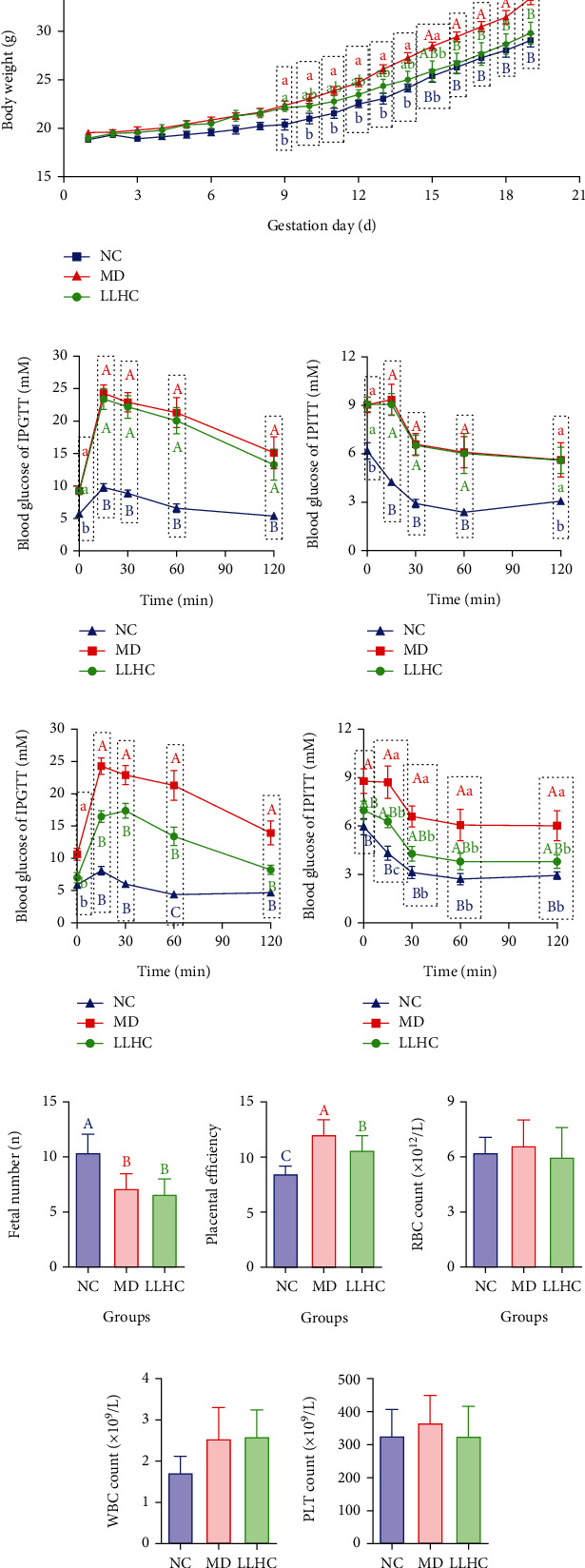
Oral administration of total water-soluble flavonoids alleviated glucose intolerance and insulin resistance in GDM mice. (a) Flow chart of the development and intervention of the GDM mouse model. (b) Changes in mouse body weight during pregnancy. (c) Blood glucose in the IPGTT before intervention with total water-soluble flavonoids (*n* = 5). (d) Blood glucose in the IPITT before intervention with total water-soluble flavonoids (*n* = 5). (e) Blood glucose in the IPGTT after intervention with total water-soluble flavonoids (*n* = 5). (f) Blood glucose in the IPITT after intervention with total water-soluble flavonoids (*n* = 5). (g) Litter sizes of fetuses at GD19 through dissection. (h) Placental efficiency (fetal weight/placental weight). (i) RBC count from routine blood tests. (j) WBC count from routine blood tests. (k) PLT count from routine blood tests. Different capital letters indicate highly significant differences (*p* < 0.01). Different lowercase letters indicate significant differences (*p* < 0.05). LLHC: *Lithocarpus litseifolius* (Hance) Chun; GDM: gestational diabetes mellitus; HFD: high-fat diet; STZ: induction and streptozocin; NC: normal control; MD: model; IPGTT: intraperitoneal glucose tolerance test; IPITT: intraperitoneal insulin tolerance test; RBC: red blood cell; WBC: white blood cell; PLT: platelet.

The animal experiments were approved by the Animal Ethical and Welfare Committee of Huaihua University (Huaihua: 2022 (A03028)). C57BL/6J mice were purchased from Changsha Tianqin Biotechnology Co., Ltd., and housed in a clean room with free access to food and water on a 12 h light/12 h dark cycle. The female mice (~9 to ~10 weeks of age and ~20 g of body weight) were mated with male mice (~11 to 14 weeks of age) at a 2:1 housing ratio to induce pregnancy. Vaginal plug detection was considered to indicate successful pregnancy, and the date of plug detection was defined as gestation day (GD) 1 [25]. On GD 1, pregnant mice were divided into three groups, namely, the normal control (NC) group (*n* = 10), GDM model (MD) group (n =10), and GDM model group treated with total water–soluble flavonoids from *L. litseifolius* (Hance) Chun (sweet tea) (LLHC) group (*n* = 10). As shown in Figure 1(a), pregnant mice in the MD and LLHC groups were fed a HFD, pregnant mice in the NC group were fed a normal diet beginning, and all the mice were weighed during pregnancy. On GDs 6–8, pregnant mice in the MD and LLHC groups were intraperitoneally injected with STZ (1% solution) at a dosage of 20 mg/kg/d (cat No. S8050, Solarbio Life Sciences, Beijing, China), and pregnant mice in the NC group were intraperitoneally injected with the same volume of normal saline. On GDs 10–16, pregnant mice in the MD and LLHC groups were intragastrically administered total water–soluble flavonoids at a dosage of 100 mg/kg/d. At GD 9 and GD 17 and at GD 10 and GD 18, the IPGTT and IPITT were carried out to monitor glucose homeostasis in all the mice. On GD 19, the experiment was terminated, and all the mice were anaesthetized and euthanized for anatomical observation and sample collection. The litter sizes of fetuses and corresponding placentas were counted and weighed. The weights of the heart, liver, spleen, lung, and kidney were also investigated to calculate the visceral indices.

### 2.3. IPGTT and IPITT

Glucose tolerance and insulin resistance were determined via the IPGTT and the IPITT, respectively. Half of the pregnant mice in each group were fasted and given free access to water for 12 h, and were then intraperitoneally injected with 20% glucose at a dose of 2 g/kg for the IPGTT (n =55). Another half of the pregnant mice in each group were fasted and given free access to water for 6 h and were then intraperitoneally injected with 7.5% U insulin at a dose of 0.75 U/kg for the IPITT (n =5). Blood samples from the tai vein were collected and directly detected via a glucometer (cat. No. GM501Air, Sinocare Inc., Changsha, China) at 0, 15, 30, 60, and 120 min after injection.

### 2.4. Histochemical Evaluation

The liver, an important organ of glucose metabolism, was collected for tissue sectioning, staining, and microexamination according to our previous study [25] and the literature [26]. Briefly, the livers were fixed in 4% PFA, dehydrated in a gradient series of ethanol, embedded in paraffin, sectioned into 5 *μ*m thick tissue, and attached to the surface of microscope slides. After baking, deparaffinization, and rehydration, the sections were subjected to hematoxylin–eosin (H&E) staining or terminal deoxyribonucleotidyl transferase (TDT)–mediated dUTP–digoxigenin nick end labelling (TUNEL) staining, followed by dehydration with a gradient series of ethanol and clearing in xylene. Then, the mounted slides were visualized and imaged through a light microscope (BX53, Olympus), and the acquired photomicrographs were analysed via Image-Pro Plus 6.0 software (Media Cybernetics).

The liver sections were evaluated by 3 pathologists from Suzhou Cancer Cell Laboratory Co., Ltd. and Huaihua University. Five different fields of microscopic view were measured for the number of inflammatory cells, ballooning area, and steatosis area of each slice. The ratio of the average area was calculated using the pathological area divided by the total measured area.

### 2.5. Analysis of Network Pharmacology

According to the literature and our previous study [24], the main active ingredients of total water–soluble flavonoids used were as candidate components on the basis of three indicators: content ≥ 0.5%, oral bioavailability (OB) ≥ 30%, and drug likeness (DL) ≥ 1.5.

The canonical simplified molecular input line entry system (SMILES) of these active ingredients was obtained through a previous study and from the PubChem database (https://pubchem.ncbi. http://nlm.nih.gov/), which were uploaded to the Swiss Target Prediction (http://targetnet. http://scbdd.com/) to predict the targets of the components. The disease targets of GDM were obtained from the GeneCards (https://www.genecards.org/) and OMIM (https://omim.org/) databases. The targets with relevance scores greater than 10 were selected. The communicative targets between the active ingredients and GDM were screened through the Venny 2.1 platform (https://bioinfogp.cnb.csic.es/tools/venny/index.html) for further analysis.

The interaction network of active ingredients with disease targets was constructed via the Network Analyser of Cytoscape 3.10.1 software, in which the nodes indicated active ingredients or disease targets, and the edges indicated the interaction between any two. The protein interactions of communicative targets involved in the prevention and treatment of GDM were searched through the interaction database platform String 12.0 (https://cn.string-db.org/). The results of the String 12.0 analysis were also visualized with Cytoscape 3.10.1 software and were screened by clustering.

The molecular interactions between the active ingredients and core targets were confirmed by AutoDock and PyMOL software. Briefly, the 3D structures of the active ingredients and core targets were downloaded from PubChem and PDB databases and were transformed as pdbqt files, following by the removal of water molecules, solvent molecules, and ions by PyMOL software. The pdbqt files were imported into AutoDock software to deduce the binding model, binding atom, and minimum binding energy. A schematic diagram of the interaction between the active ingredients and core targets was constructed and visualized by AutoDock and PyMOL software.

The signalling pathways of the main anti-GDM components were analysed and visualized by Gene Ontology (GO) enrichment and Kyoto Encyclopedia of Genes and Genomes (KEGG) enrichment in the Metascape platform (http://Metascape.org/gp/index.html) and the Cytoscape plugin ClueGO, with *p* < 0.05 and kappa scores ≥ 0.6.

### 2.6. Statistical Analysis

Statistical analyses were performed via one-way ANOVA with a post hoc multiple comparison (Bonferroni) test after normal distribution tests, which were performed via the Statistical Package for Social Science (SPSS, Version 19.0; SPSS Inc, Chicago, IL, United States). The final data are shown as the mean ± standard deviation (SD), with differences considered significant at *p* < 0.05 or *p* < 0.01.

## 3. Results

### 3.1. Oral Administration of Total Water–Soluble Flavonoids Alleviated Glucose Intolerance and Insulin Resistance in GDM Patients

The increase in mouse body weight in the LLHC group was significantly lower than that in the MD group and tended to increase in the NC group (Figure 1(b)). These data suggested that the oral administration of total water–soluble flavonoids was beneficial for controlling excessive increases in body weight.

Before intervention with total water–soluble flavonoids, both glucose tolerance and insulin sensitivity were impaired in the LLHC and MD groups (Figure 1(c) and 1(d)) at GDs 9 and 10. However, they were regular in the NC group. These data demonstrated the successful development of the GDM mouse model.

Compared with those in the MD group, after intervention with total water–soluble flavonoids from GDs 10–16, both glucose tolerance and insulin sensitivity were partly rescued in the LLHC group, but the glucose tolerance and insulin sensitivity were lower than those in the NC group (Figure 1(e) and 1(f)) at GDs 17 and 18. These data revealed that total water–soluble flavonoids stabilized blood glucose when applied to GDM mice.

Compared with those in the NC group, the litter sizes of fetuses in both the MD and LLHC groups were significantly decreased. However, there were no significant differences in the litter size of foetuses between the MD and LLHC groups (Figure 1(g)).

Foetal and placental weights were also investigated for calculation of placental efficiency (foetal weight/placental weight), which revealed that the placental efficiency in the MD group was the highest among those in the NC, MD, and LLHC groups. These parameters were greater in the LLHC group than in the NC group (Figure 1(h)). These data indicated that high placental efficiency was induced when GDM occurred and was partly inhibited by the oral administration of total water–soluble flavonoids.

Routine blood tests revealed that the RBC, WBC, and PLT counts were not significantly different among the NC, MD, and LLHC groups (Figures 1(i), 1(j), and 1(k)). These data suggested that the oral administration of total water–soluble flavonoids did not induce systemic blood changes.

### 3.2. Oral Administration of Total Water–Soluble Flavonoids Inhibited Liver Cell Apoptosis to Improve Glucose Homeostasis in GDM Mice

**Figure 2:**
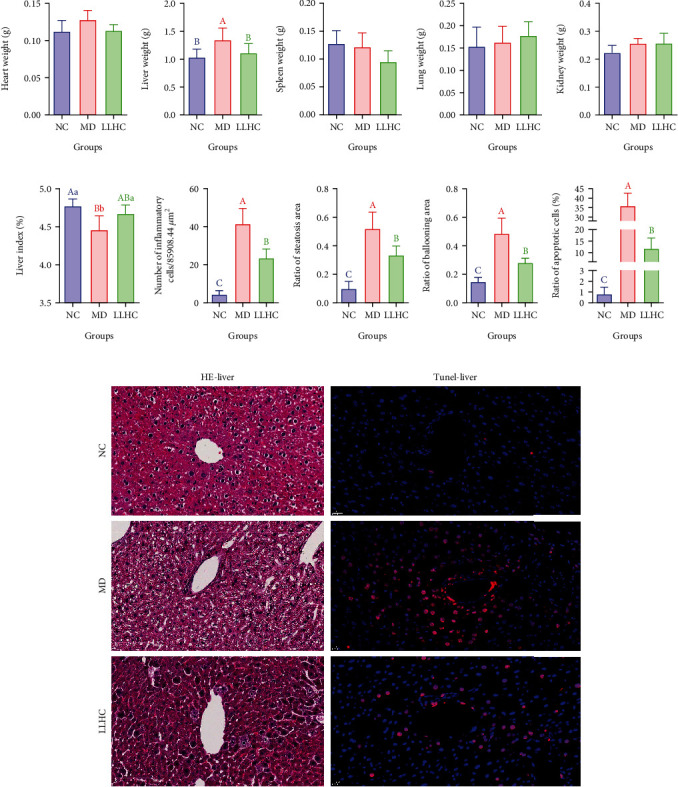
Oral administration of total water-soluble flavonoids inhibited liver cell apoptosis to improve glucose homeostasis in GDM mice. (a) Heart weight. (b) Liver weight. (c) Spleen weight. (d) Lung weight. (e) Kidney weight. (f) Liver indices (liver weight/body weight). (g) Number of inflammatory cells per 85908.44 *μ*m^2^. (h) Ratios of the steatotic area of the liver sections. (i) Ratios of the ballooning area of the liver sections. (j) Ratio of apoptotic cells (%). (k) H&E and TUNEL staining of liver sections. Different capital letters indicate highly significant differences (*p* < 0.01). Different lowercase letters indicate significant differences (*p* < 0.05). LLHC: *Lithocarpus litseifolius* (Hance) Chun; GDM: gestational diabetes mellitus; NC: normal control; MD: model; H&E: hematoxylin–eosin; TUNEL: terminal deoxyribonucleotidyl transferase (TDT)–mediated dUTP–digoxigenin nick end labelling.

The internal organs were weighed and compared. There were no significant differences in heart, spleen, lung, or kidney weights among the NC, MD, and LLHC groups (Figures 2(a), 2(c), 2(d), and 2(e)). However, liver weight was significantly greater in the MD group than in the NC group. There was no significant difference in liver weight between the LLHC and NC groups, but liver weight was significantly lower in the LLHC group than in the MD group. Moreover, the liver indices (liver weight/body weight) were significantly lower in the MD group than in the NC group. The liver indices of the LLHC- and NC-treated mice were not significantly different, but the liver indices of the LLHC-treated mice were significantly greater than those of the MD-treated mice (Figure 2(b) and 2(f)).

H&E staining of liver tissue sections from the LLHC group revealed that the number of inflammatory cells was significantly lower than that from the MD group but greater than that from the NC group (Figures 2(g) and 2(k)). There were many small holes, which are the indicators of adipose degeneration, in the liver sections from the MD group but few holes in those from the LLHC group and fewer small holes in those from the NC group. After detection and statistical analysis, the ratios of steatosis area and ballooning area in the liver sections from the LLHC group were significantly lower than those in the liver section from the MD group but greater than that from the NC group (Figures 2(h), 2(i), and 2(k)).

TUNEL staining of the liver tissue sections revealed that the apoptotic cells were red. The number of apoptotic cells was significantly greater in the liver cell sections from the MD group than in those from the NC group. The number of apoptotic cells was also significantly greater in the liver cell sections from the LLHC group than in those from the NC group but was significantly lower than that in the liver cell sections from the MD group. The ratio of apoptotic cells (the number of apoptotic cells/the number of normal cells) was greatest in the MD group and greatest in the LLHC group among the NC, MD, and LLHC groups (Figure 2(j) and 2(k)).

These data suggested that the oral administration of total water–soluble flavonoids partially inhibited the apoptosis of liver cells and rescued liver glucose metabolism and homeostasis.

### 3.3. Multiple Targets and Signalling Pathways Were Involved in the Process by Which Total Water–Soluble Flavonoids Protect Against GDM

**Figure 3:**
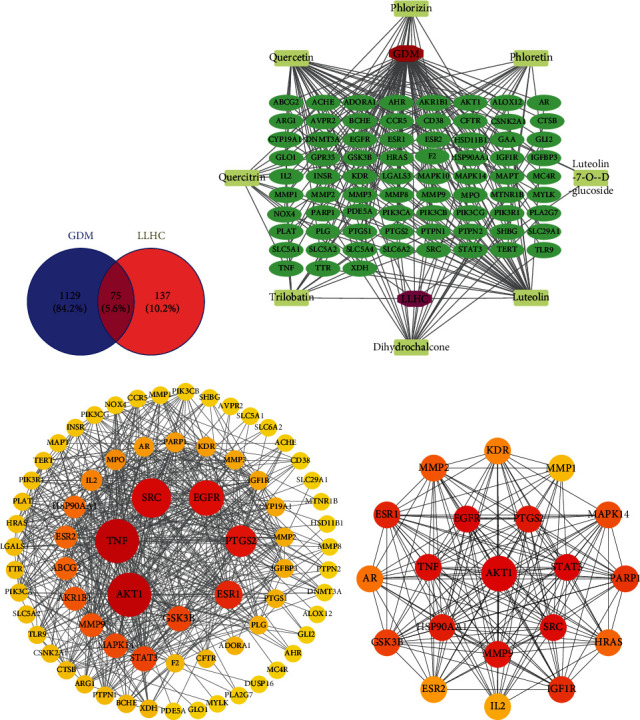
Multiple targets were involved in the process by which total water-soluble flavonoids protect against GDM. (a) The targets of GDM and the active ingredients identified in the GeneCards and OMIM databases, the Swiss Target Prediction System, and the communicative targets were screened through the Venny 2.1 platform. (b) The interaction network of GDM, active ingredients, and communicative targets was constructed with the Network Analyser of Cytoscape 3.10.1 software. (c) The interaction relationships of the targets were determined through the interaction database platform String 12.0. (d) The interaction relationships of the Top 20 scores (2.69e + 08) ranked by the MCC method.

To further investigate the potential mechanism of total water–soluble flavonoids against GDM, a total of 1204 GDM targets were identified from the GeneCards and OMIM databases. A total of 215 component targets were identified from the Swiss Target Prediction system. A total of 75 communicative targets associated with both GDM and total water–soluble flavonoids were identified (Figure 3(a)). All of these communicative targets were related to both the pharmacodynamic substances of total water–soluble flavonoids and the disease occurrence of GDM, including 85 nodes and 598 edges (Figure 3(b)). Analysis of protein interactions revealed 72 nodes and 292 edges with an average node degree of 15.9 and an average local clustering coefficient of 0.628 (Figure 3(c)). The communicative targets with the Top 20 scores (2.69e + 08), ranked by the maximal clique centrality (MCC) method, were selected for further analysis. The results revealed 20 nodes and 169 edges. These genes were AKT1, STAT3, EGFR, SRC, TNF, MMP9, PTGS2, HSP90AA1, ESR1, IGF1R, PARP1, GSK3B, MAPK14, MMP2, HRAS, AR, KDR, ESR2, IL2, and MMP1 in descending order (Figure 3(d)).

**Figure 4:**
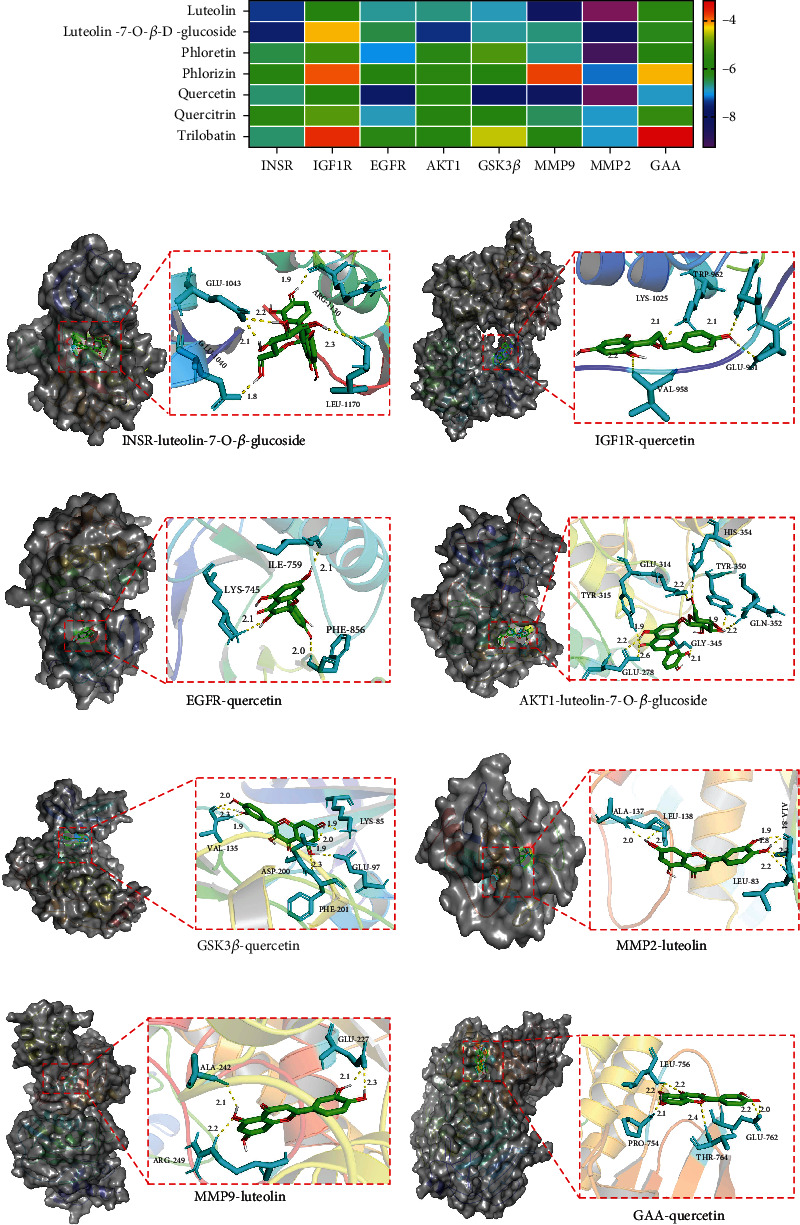
The interactions between the core targets and active ingredients were confirmed by molecular docking and visualization. (a) Heatmap analysis of minimum binding energy of the molecular docking fraction. (b) Docking poses and interaction of INSR with luteolin-7-O-*β*-glucoside. (c) Docking poses and interactions of IGF1R with quercetin. (d) Docking poses and interactions of EGFR with quercetin. (e) Docking poses and interactions of AKT1 with luteolin-7-O-*β*-glucoside. (f) Docking poses and interactions of GSK3*β* with quercetin. (g) Docking poses and interactions of MMP2 with luteolin. (h) Docking poses and interactions of MMP9 with luteolin. (i) Docking poses and interactions of GAA with quercetin.

These eight targets, including INSR, IGF1R, EGFR, AKT1, GSK3*β*, MMP9, MMP2, and GAA, were considered closely associated with total water–soluble flavonoids against GDM and were selected to investigate their molecular interactions. Seven ingredients were used as ligands for molecular docking. The binding energies for molecular docking were recorded (Figure 4(a)). When the molecular docking binding energy is < 0, the two molecules have spontaneous binding ability; when the molecular docking binding energy is < −1.2 kcal/mol (−5.0 kJ/mol), the two molecules are well bound [27]. On the basis of these models, the best interaction sites and action sites between the core targets and active ingredients were recorded, including INSR with luteolin-7-O-*β*-glucoside (Figure 4(b)), IGF1R with quercetin (Figure 4(c)), EGFR with quercetin (Figure 4(d)), AKT1 with luteolin-7-O-*β*-glucoside (Figure 4(e)), GSK3*β* with quercetin (Figure 4(f)), MMP2 with luteolin (Figure 4(g)), MMP9 with luteolin (Figure 4(h)), and GAA with quercetin (Figure 4(i)). Therefore, quercetin, luteolin, and luteolin-7-O-*β*-glucoside had the greatest effects on the core targets of GDM.

### 3.4. Total Water–Soluble Flavonoids Improve Glucose Homeostasis in GDM Through Six Main Signalling Pathways Around the AKT1 Core Axis

**Figure 5:**
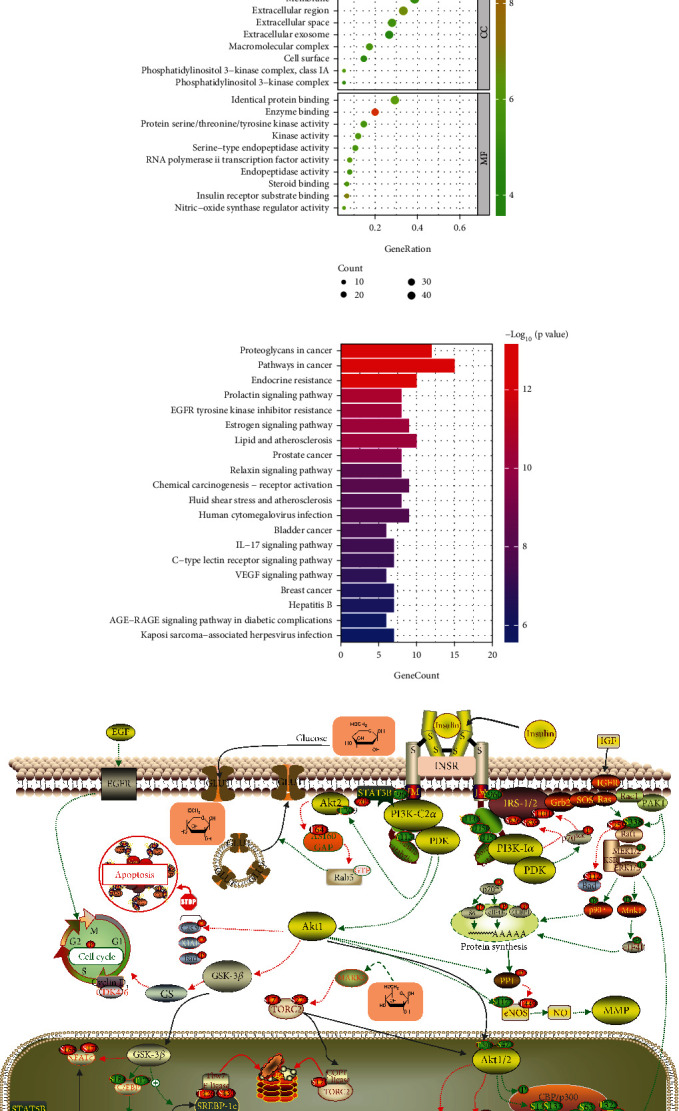
Total water-soluble flavonoids were deduced to improve glucose homeostasis in GDM through 6 main signalling pathways around the AKT1 core axis. (a) The signalling pathways associated with the main anti-GDM components were analysed and visualized by GO enrichment. (b) The signalling pathways of the main anti-GDM components were analysed and visualized via KEGG enrichment. (c) In the process of total water-soluble flavonoids improving GDM glucose homeostasis, quercetin affects AKT1, EGFR, SRC, MMP9, IGF1R, PARP1, GSK3B, MMP2, KDR, and ESRS. Luteolin affects AKT1, EGFR, SRC, ESR1, IGF1R, GSK3B, IGF1R, KDR, and ESR2.

All of these important targets were involved in 30 signalling pathways. These signalling pathways, including the apoptotic process signalling pathway, insulin receptor signalling pathway, steroid binding pathway, and insulin receptor substrate binding pathway, may be directly involved in GDM occurrence and outcome. These signalling pathways were considered upstream regulatory signals and included signal transduction, gene expression, protein kinase B signalling, the MAPK cascade, MAP kinase activity, phosphatidylinositol 3-kinase complex, and nitric oxide synthase regulation activity (Figure 5(a)). These signalling pathways were associated with 20 kinds of pathogenesis, in which endocrine resistance, the estrogen signalling pathway, the VEGF signalling pathway, and the AGE-RAGE signalling pathway in diabetic complications were directly or indirectly involved in GDM occurrence and outcome (Figure 5(b)).

As shown in Figure 5(c), the binding of Ins to INSR and the binding of IGF1 to IGFR act on IRS-1 to activate PI3K. Moreover, TNF*α* binds to TNFR to act on JNK1 to inhibit IRS-1, and IL2 binds to IL2R to act on STAT3 and SOS3 to inhibit IRS-1. There is a balance between Ins and TNF signalling and between IBS and IL2 signalling. EGF binding to EGFR also acts on PI3K, and E2 binding to the ER acts on SRC for PI3K activation. These signals of PI3K activation are transferred to AKT1 through the second messengers PIP3 and PKD1. AKT1 signalling blocks the inhibitory effect of GSK3*β* on GS, and activated GS enables glycogen synthesis to reduce the production of endogenous glucose and alleviate insulin resistance. AKT1 signalling also inhibits GLUT through blocking the inhibitory effects of AS160 and GAP on Rab5 to reduce the intake of exogenous glucose. Moreover, AKT1 signal stops the apoptotic signal in liver cells. All of the above signals benefit the cell cycle progression and survival of liver cells. After GSK3*β* is inhibited, NFATc1 dissociates from C/EBP and reacts with STAT to increase the transcription and translation of glycometabolism genes. AKT1 acts on CREB and TORC2 through CBP/p3000 to inhibit the transcription and translation of blood glucose-related genes. However, HSP90 acts on eNOS to promote NO production, which reduces liver cell fibrosis. In the process by which total water–soluble flavonoids improve glucose homeostasis, quercetin (acting on IGF1R, EGFR, GSK3B, and GAA), luteolin-7-O-*β*-glucoside (acting on INSR and AKT1), and luteolin (acting on MMP2 and MMP9) may act as medicinal substances.

## 4. Discussion

The complex etiology of GDM is not entirely clear. There may be an accumulation of pathogens during pregestation and an outbreak of symptoms during pregnancy induced by changes in nutritional status and hormonal levels. Therefore, preventive intervention ahead of pregnancy or adaptive intervention during pregnancy may have good effects.

These potential practices were identified in people in these regions who drink *L. litseifolius* (Hance) Chun (sweet tea) and have a relative fit of body weight and a relative incidence rate of GDM (3.8% through investigation). The main event in the consumption of *L. litseifolius* (Hance) Chun (sweet tea) is water-soluble substance ingestion. In our previous study, we detected many water-soluble flavonoids in aqueous extracts of *L. litseifolius* (Hance) Chun (sweet tea) [24], which was also in accordance with other studies [28, 29]. Moreover, some studies have shown that flavonoids have a function against GDM [30, 31]. However, the water solubility and bioavailability of flavonoids are still obstacles to their medicinal application. In this study, the total flavonoids, which were aqueously extracted from *L. litseifolius* (Hance) Chun (sweet tea), exhibited good water solubility and bioavailability. These properties of total water–soluble flavonoids may be advantageous for the application of anti-GDM agents.

Chinese herbal medicines are relatively complex and usually play roles through multiple targets and multiple pathways [32]. In this study, total water–soluble flavonoids were also complex and may exert overall regulatory and synergistic effects on blood glucose homeostasis in GDM patients. Indeed, in the analysis of potential mechanisms, we found that multiple targets and multiple signalling pathways were involved in the process by which total water–soluble flavonoids protect against GDM, including promoting the transformation between glucose and glycogen to decrease the production of endogenous glucose and excessive aggregation of blood glucose, promoting cell metabolism and inhibiting cell apoptosis to increase insulin sensitivity. These data suggest that the effects of total water–soluble flavonoids against GDM are comprehensive. A recent study revealed that AKT1 is an important pathological and therapeutic target of GDM [33]. So the interaction of active ingredient with AKT1 may play central roles in the process by which total water–soluble flavonoids protect against GDM.

In previous studies, quercetin was used as a functional food additive that has been linked to a lower risk of GDM, has beneficial effects on pregnant mothers and their fetuses [34], and attenuates histological abnormalities associated with GDM via the upregulation of placental adiponectin and adiponectin receptors [31]. The estrogen–like chemical structure of quercetin has a high affinity for the estrogen receptor, suggesting the likely mechanism of total water–soluble flavonoids against GDM. Luteolin has also been reported to have an antidiabetic function that was linked with increased expression of PPAR*γ* and GLUT [35]. Although there is no direct evidence of references on the effects of luteolin against GDM, it is not difficult to understand according to the homology of GDM drug use with diabetes. Both quercetin and luteolin have been shown to enhance glucose tolerance and insulin sensitivity in diabetes studies [36, 37]. Even so, further research is needed on the function and mechanism of quercetin and luteolin in the treatment of GDM.

During the process of modelling and drug intervention, some fetuses may be lost due to the influence of STZ or intervention drugs. Therefore, we compared the number of fetuses to ensure that the intervention medication did not cause a decrease in the number of fetuses. Our data suggested that foetal loss was induced by GDM model construction rather than by the oral administration of total water–soluble flavonoids.

In conclusion, the oral administration of total water–soluble flavonoids can alleviate glucose intolerance and insulin resistance and inhibit liver cell apoptosis. The main active ingredients, quercetin and luteolin, act on GAA through the signalling pathways of the AKT core axis.

## Data Availability

The data that support the findings of this study are available from the corresponding authors upon reasonable request.

## Nomenclature

GDM: gestational diabetes mellitus
GO: Gene Ontology
H&E: hematoxylin–eosin
HFD: high-fat diet
IPGTT: intraperitoneal glucose tolerance test
IPITT: intraperitoneal insulin tolerance test
KEGG: Kyoto Encyclopedia of Genes and Genomes
LLHC: *Lithocarpus litseifolius* (Hance) Chun (sweet tea)
MD: model
NC: normal control
PLT: platelet
RBC: red blood cell
SMILES: simplified molecular input line entry system
STZ: induction and streptozocin
TUNEL: terminal deoxyribonucleotidyl transferase (TDT)–mediated dUTP–digoxigenin nick end labelling
WBC: white blood cell
